# Metabotropic Glutamate Receptor 3 Is Associated with Heroin Dependence but Not Depression or Schizophrenia in a Chinese Population

**DOI:** 10.1371/journal.pone.0087247

**Published:** 2014-01-31

**Authors:** Wei Jia, Rui Zhang, Bin Wu, Zun-xiao Dai, Yong-sheng Zhu, Ping-ping Li, Feng Zhu

**Affiliations:** 1 Methadone Maintenance Treatment Clinic, Xi’an Mental Health Center, Xi’an, Shannxi, China; 2 Forensic Department, Xi’an Jiaotong University College of Medicine, West Yanta Road, Xi’an, Shaanxi, China; 3 Center for Translational Medicine, The First Affiliated Hospital of Xi’an Jiaotong University College of Medicine, Xi’an, Shaanxi, China; Sudbury Regional Hospital, Canada

## Abstract

Metabotropic glutamate receptor subtype 3 (mGluR3, encoded by *GRM3*) plays important roles in the pathophysiology of schizophrenia, depression, and drug dependence. *GRM3* polymorphisms were reported to be associated with prefrontal activity, cognitive shifting, and memory capability in healthy subjects, as well as susceptibility to schizophrenia and depression. The goal of this study was to replicate the association of *GRM3* with schizophrenia and depression and to explore *GRM3’s* potential association with heroin dependence (HD) in a Chinese population. Seventeen SNPs throughout the *GRM3* gene were genotyped using MALDI-TOF within the MassARRAY system, and the allele and genotype distributions were compared between 619 healthy controls and 433 patients with schizophrenia, 409 patients with major depression, and 584 unrelated addicts. We found that *GRM3* polymorphisms modulate the susceptibility to HD but do not significantly influence the risk for schizophrenia or depression. An increased risk of HD was significantly associated with the minor alleles of two *GRM3* SNPs, including the T allele of rs274618 (Odds ratio (OR) = 1.631, 95% confidence interval (95%CI): 1.317–2.005), the T allele of rs274622 (OR = 1.652, 95% CI: 1.336–2.036), compared with the major alleles. The addicts carrying the minor allele of rs274618 or rs274622 had a shortened duration for transition from first use to dependence (DTFUD) in comparison to homozygote for major allele (*P*<0.0001 for each SNP using log rank test). Additionally, a 6-SNP haplotype within 5′ region of the *GRM3* including the minor alleles of the two aforementioned SNPs was significantly associated with an increased risk of HD (*P* = 0.00001, OR = 1.668, 95% CI: 1.335–2.084). Our data indicated that *GRM3* polymorphisms do not contribute to genetic susceptibility to schizophrenia and depression, but they confer an increased risk of HD in a Chinese population.

## Introduction

Glutamate is the primary excitatory neurotransmitter in the brain that mediates the synaptic plasticity specifically linked with adaptive behavior [1], [2]. Accumulating lines of evidence implicate abnormalities in glutamate neural transmission in the pathophysiology of multiple brain diseases, including schizophrenia [3], depression [4], [5], [6], and addiction [7]. Moreover, genetic variations in glutamate pathway-related genes are associated with cognitive function, response to medication and vulnerability to these diseases [8], [9], [10], [11]. Glutamate activates various receptors, including N-methyl-d-aspartate (NMDA) receptor, metabotropic glutamate receptors (mGluRs) and α-amino-3-hydroxy-5-methyl-4-isoxazole propionate (AMPA) receptor. mGluRs mediate signal transduction through G-protein second messenger systems, whereas NMDA and AMPA receptors act as ligand-gated ion channels. Previous studies suggest mGluRs “fine tune” glutamate transmission, whereas ionotropic receptors regulate large-scale glutamate fluctuations in the brain [12]. mGluRs have also been found to play important roles in the pathophysiology of schizophrenia [13], depression [6], and addiction [14].

A total of eight mGluRs encoded by metabotropic glutamate receptor genes (*GRMs*) are classified into three groups (I–III) according to sequence homology, signal transduction pathways, and pharmacological properties [15]. The group II mGluRs, including mGluR2 and mGluR3 (mGluR 2/3), are implicated in the etiology of schizophrenia. Agonists of group II mGluRs have been shown to ameliorate the psychotic effects induced by phencyclidine and block the behavioral and prefrontal cognitive abnormalities induced by the psychosis-producing drug ketamine, suggesting that a possible deficit in mGluR3 functions in psychotic disorders such as schizophrenia [16]. *GRM3* is located at human chromosome 7q21.1–q21.2 [17] and has been shown to be a promising candidate gene for schizophrenia. Three previous studies have reported positive associations between three single nucleotide polymorphisms (SNPs), rs1468412, rs6465084, and rs2299225, in *GRM3* and schizophrenia in Japanese [18], European American [8], and Chinese populations [19]. However, these positive findings were not reproducible in other independent studies [20], [21], [22], [23], [24]. Interestingly, recent studies found that *GRM3* polymorphisms (rs1989796 and rs1476455) were associated with symptoms of refractory global psychosis in patients with schizophrenia [9]. These inconsistent findings highlight the need to elucidate the role of *GRM3* in genetic predisposition to schizophrenia.

*GRM3* is also a promising candidate gene for depression. The antagonists of group II mGluRs, such as MGS0039 and LY341495, exert dose-dependent antidepressant-like effects in murine models of depression, including the forced swim and tail suspension tests [25]. mGluR3 is highly expressed in the forebrain and limbic areas, which likely participate in the emotion process [26], [27]. Furthermore, mGluR3 expression significantly decreased in the perirhinal cortex of patients with major depressive disorder (MDD) or bipolar disorder [28], suggesting that disturbance in mGluR3-mediated synaptic transmission may be an important factor in the pathophysiology of depression. Tsunoka et al. [10] first reported that rs6465084 in *GRM3* is significantly associated with MDD in a Japanese population. Only one *GRM3* SNP was analyzed in their study; therefore, further studies analyzing multiple loci should be performed to replicate their findings and evaluate the effects of other variations of *GRM3* on the risk of depression.

*GRM3* is involved in the development of addiction [14]. Repeated exposure to drugs of abuse, including cocaine [29], [30], [31], morphine [32], [33] and nicotine [34], [35], causes neuroadaptations in mGluR 2/3, such as altered receptor density, Gi signaling and receptor-dependent plasticity. Animal studies show that mGluR 2/3 regulate both reward processing and drug seeking. Cocaine-induced dopamine release in the rat nucleus accumbens (NAc) [36] and self-administration of cocaine and nicotine in rats [37], [38] were attenuated by mGluR 2/3 agonists, such as LY379268, 2-PMPA, and LY314582, suggesting that these receptors have negative regulatory effects on the reward process. Systemic injections of the agonists for mGluR 2/3 blocked drug- or cue-elicited relapse to cocaine [37], [39], heroin [40], [41], nicotine [38] and alcohol [42] after self-administration of the drug. These findings suggested that activation of mGluR 2/3 negatively regulates drug-seeking. Although the important roles of mGluR 2/3 in addiction have been established [14], the influence of genetic polymorphisms in *GRM3* on an individual’s susceptibility to drug dependence has not been reported. Recently, we found that the duration for transition from first use to dependence (DTFUD), measuring the efficiency of transition to dependence, is a new phenotype to define inherited susceptibility to opioid dependence. And genetic factors can alter the risk for substance dependence via affecting the efficiency of transition to dependence [43]. The significant associations of *GRM3* polymorphisms with prefrontal activity (measured by N-acetylaspartate/creatine levels) [44], cognitive set shifting [45], memory capability in healthy volunteers [46] and the risk of schizophrenia and depression in some studies [8], [10] promoted us to further evaluate the role of *GRM3* polymorphisms in heroin dependence (HD).

## Experimental Procedure

### 1. Samples

A total of 433 unrelated patients with schizophrenia (228 males and 205 females, mean age of onset: 29.5±9.2 years) and 409 unrelated patients with major depression (185 males and 224 females, mean age of onset: 34.2±10.5 years) were recruited from the mental health center of the First Affiliated Hospital, Xi’an Jiaotong University and the Xi’an Mental Health Center. A total of 584 unrelated heroin addicts receiving treatment in the Methadone Maintenance Treatment Program at the Xi’an Mental Health Center were recruited for the present study (486 males and 98 females, mean age of onset: 25.4±7.3 years). All patients were interviewed independently by two psychiatrists applying DSM-IV diagnostic criteria for schizophrenia, depression and opioid dependence. Patients with two or more types of mental illness or substance dependence were excluded.

The controls consisted of 619 unrelated healthy people (409 males and 210 females, mean age: 36.1±10.3 years) who underwent psychological counseling in our hospital. A series of questionaires were completed by them to confirm that each control had no mental illness or substance dependence. All participants were of Han Chinese descent. All subjects participated voluntarily, were capable of consenting for themselves and signed written informed consent prior to the enrollment. The written informed consent mainly included the title, purpose, methods and potential scientific significance of research, the name and contact information of researchers involved, the duty and right of participants involved, and the statement about how to protect the privacy of participants. This research was approved by the local medical ethics committee. All subjects completed written informed consent forms.

### 2. DTFUD Investigation

The method to obtain DTFUD has been described in our previous study [43]. In brief, the duration from the initial opioid use to first occurrence of the dependence syndrome according to DSM-IV was assessed by an interviewer and blindly verified by an independent psychiatrist using medical records and information provided by first-degree relatives, only the consistent data from these three sources were included in analysis. Subjects who used drugs less than three times per week in the first month or could not obtain drugs for more than one week were excluded from the DTFUD analysis.

### 3. Selection of Polymorphisms

**Figure 1:**
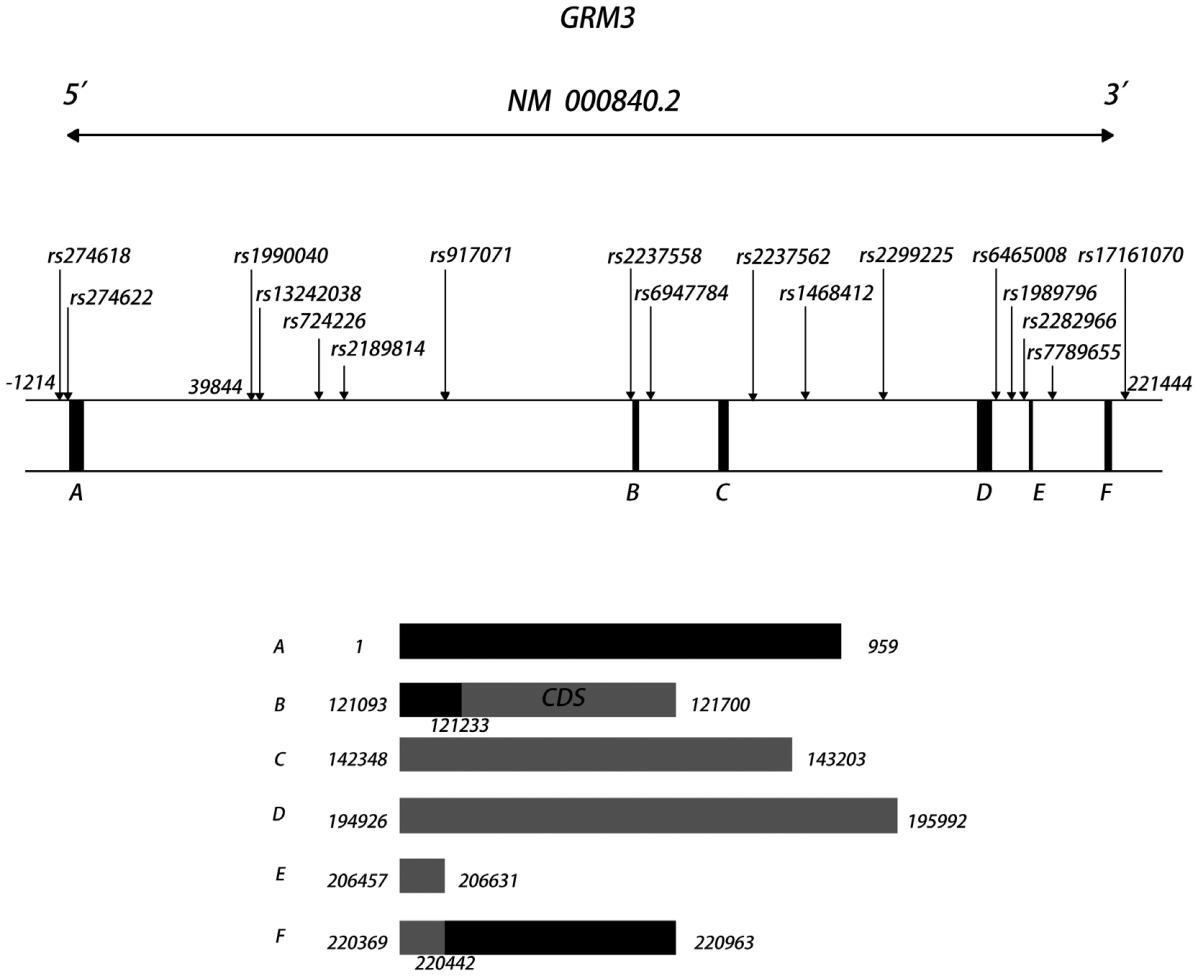
Gene structure of human *GRM3* and the relative positions of the 17 SNPs used in our study. The schematic diagram of *GRM3* gene is based on NCBI reference sequences NM 00084.2 and marks exons by the black bars orderly nominated A–F. The bands with serial number A–F below gene chart show the start and end position of corresponding exon in their left and right terminals. The black parts of these bands represent untranslated sequence, and the gray parts of these bands represent coding DNA sequence (CDS).

The genomic sequence of *GRM3* spans 221 kb. Most previous studies only analyzed a few SNPs, which are insufficient to cover the gene locus. A total of 17 genetic polymorphisms throughout the *GRM3* gene and the 5′ and 3′ flanking regions were selected for the present study. The selected polymorphisms included SNPs that had positive associations with schizophrenia and memory in previous studies [9], [18], [19], [46] and most tagSNPs that were revealed by genotype data of the Han Chinese population from HapMap. The identity of these polymorphisms and their relative location in the gene are shown in Fig. 1.

### 4. Genotyping

Genomic DNA was extracted from blood leukocytes using TIANamp Blood Genomic DNA Purification Kit (Tiangen Biotech, Beijing, China), according to the manufacturer’s protocol. The selected SNPs were genotyped in patients and controls by using MALDI-TOF within the MassARRAY system (Sequenom Inc., San Diego, CA, USA). Primers used in MALDI-TOF are shown in Table S1.

### 5. Statistical Analysis

We calculated the statistical power as previously described [47]. Differences between the patients and controls in the frequency of the alleles, genotypes and haplotypes were evaluated by chi-square analysis or Fisher’s exact test. Unconditional logistic regression was used to calculate the odds ratio (OR) and 95% confidence interval (CI), which were used to evaluate the strength of association between each locus and the presence of disease. The potential correlation of DTFUD with genotype and allele was evaluated using Kaplan-Meier survival analysis with log rank test as describled in our previous study [43]. All *P* values presented were two sided, and a level of *P*<0.05 was considered statistically significant. To avoid spurious association caused by multiple tests, the false positive report probability (FPRP) was calculated according to the method described by Wacholder et al. [48]. We set 0.2 as an FPRP threshold and assigned a prior probability of 0.01 to detect an OR of 1.50 (for risk effects) or 0.67 (for protective effects) for an association with the SNPs under investigation. Only significant results with an FPRP value <0.2 were considered noteworthy associations. All statistical analyses were carried out using SPSS 16.0 software (SPSS Inc., Chicago, IL, USA). Haploview 4.2 [49] was used to calculate pair-wise linkage disequilibrium (LD) statistics (D′ and r^2^), to calculate haplotype frequency and to evaluate deviation from Hardy–Weinberg equilibrium (HWE).

## Results

**Figure 2:**
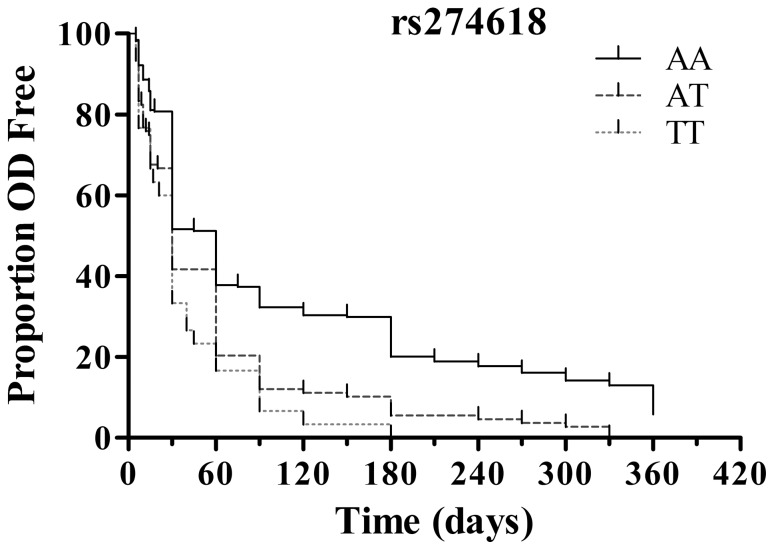
Kaplan–Meier survival curves representing probability that individuals have not experienced dependence over the period of time following first opioid use. Total addicts stratified by genotype of rs274618.

More than 99% of the samples were genotyped successfully for each SNP, and the replicate experiment for the randomly selected 650 samples (31.8%) acquired completely consistent genotype data with the original analysis. No significant deviation from HWE was found for any of the SNPs in the controls and patients with depression or schizophrenia. In heroin addicts, four SNPs (rs274618, rs274622, rs3242038 and rs724226) significantly deviated from HWE (*P*<0.05). LD analysis in healthy controls revealed that 17 SNPs were located in four separate haplotype blocks; the haplotype structure and pair-wise LD values (r^2^) are shown in Fig. 2. The structure of the haplotype blocks and pair-wise LDs calculated from each patient group were roughly similar to those in controls (data not shown).

Given an OR of 1.5 at a nominal *P* = 0.05 for genotype frequencies ranging from 0.15 to 0.30, the statistical power of our study to detect an association of *GRM3* with schizophrenia, HD, and major depression was estimated at 70.0–86.7%, 76.5–91.5%, and 68.6–85.6%, respectively. The allelic and genotypic frequencies of the 17 *GRM3* SNPs in the controls and patients with different diseases are shown in Table 1. In heroin addicts, six SNPs (rs274618, rs274622, rs1990040, rs13242038, rs724226, and rs2189814) exhibited nominally significantly different genotypes and/or allele distributions compared with the controls (*P*<0.05). Three SNPs remained robust after evaluation by FPRP and included rs274618 and rs274622. Compared with the controls, the minor alleles of these three SNPs were over-represented in heroin addicts. Additionally, logistic regression analysis revealed that the minor alleles were significantly associated with an increased risk of HD after adjustment for demographic factors, including age, gender, education level, unemployment, and life history with family (for the T allele of rs274618, OR = 1.631, 95% CI: 1.317–2.005 and FPRP = 0.002; for the C allele of rs274622, OR = 1.652, 95% CI: 1.336–2.036 and FPRP = 0.001; for the A allele of rs724226, OR = 1.579, 95% CI: 1.271–1.964 and FPRP = 0.012). The probability for emergence of dependence at a certain time after first opioid use was compared between the subjects with different genotype of 17 *GRM3* SNPs. We found that the subjects carrying the minor allele of rs274618 or rs274622 had shorter DTFUD in comparison to homozygote for major allele (*P*<0.0001 for each SNP using log rank test, Fig. 2).

**Table 1:** Genotypic and allelic frequencies of *GRM3* polymorphisms in the controls and patients with different diseases.

	Controls	Sz			HD			Dep		
Variable	(n = 619 )	(n = 433)	*P*-value^a^	OR^b^, 95% CI	(n = 584)	*P*-value	OR, 95% CI	(n = 409)	*P*-value	OR, 95% CI
	N, %	N, %			N, %			N, %		
rs274618			0.025			3×10^−5^			0.653	
AA	459, 74.2	298, 69	0.066	1.29, 0.988∼1.691	381, 65.2	0.001	**1.529, 1.196∼1.96**	306, 74.8	0.811	0.962, 0.721∼1.289
AT	144, 23.3	110, 25.5	0.412	1.13, 0.851∼1.5	158, 27.1	0.130	1.223, 0.945∼1.585	89, 21.8	0.573	0.915, 0.682∼1.241
TT	16, 2.6	24, 5.6	0.013	2.22, 1.168∼4.227	45, 7.7	5×10^−5^	3.142, 1.755∼5.627	14, 3.4	0.435	1.337, 0.649∼2.767
Per T allele	176, 14.2	158, 18.3	0.012	1.353, 1.071∼1.71	248, 21.2	6×10^−6^	**1.631, 1.317∼2.005**	117, 14.3	0.956	1.005, 0.779∼1.298
rs274622			0.024			2×10^−5^			0.666	
TT	459, 74.4	297, 69.1	0.059	1.3, 0.99∼1.706	380, 65.1	0.0004	**1.563, 1.213∼2.002**	304, 74.9	0.862	0.977, 0.732∼1.299
TC	142, 23	109, 25.3	0.384	1.132, 0.852∼1.514	159, 27.2	0.092	1.25, 0.961∼1.625	88, 21.7	0.616	0.925, 0.684∼1.247
CC	16, 2.6	24, 5.6	0.013	2.223, 1.169∼4.236	45, 7.7	5×10^−5^	3.141, 1.756∼5.613	14, 3.4	0.428	1.344, 0.648∼2.776
Per C allele	174, 14.1	157, 18.3	0.010	1.365, 1.075∼1.721	249, 21.3	3×10^−6^	**1.652, 1.336∼2.036**	116, 14.3	0.906	1.01, 0.787∼1.306
rs1990040			0.164			0.010			0.849	
GG	299, 48.3	193, 44.6	0.233	1.162, 0.909∼1.491	251, 43	0.064	1.239, 0.988∼1.558	205, 50.1	0.568	0.932, 0.726∼1.19
GA	253, 40.9	177, 40.9	0.999	1.001, 0.78∼1.284	236, 40.4	0.871	0.978, 0.779∼1.233	161, 39.4	0.629	0.938, 0.728∼1.213
AA	67, 10.8	63, 14.5	0.071	1.406, 0.974∼2.031	97, 16.6	0.003	1.638, 1.177∼2.297	43, 10.5	0.875	0.972, 0.646∼1.454
Per A allele	387, 31.3	303, 35	0.073	1.179, 0.983∼1.42	430, 36.8	0.004	1.284, 1.081∼1.518	247, 30.2	0.609	0.952, 0.784∼1.157
rs13242038			0.305			0.035			0.011	
CC	546, 88.9	376, 88.3	0.741	1.073, 0.721∼1.573	536, 92.6	0.030	0.644, 0.43∼0.963	380, 93.8	0.008	0.528, 0.324∼0.849
CT	64, 10.4	43, 10.1	0.863	0.969, 0.643∼1.449	37, 6.4	0.012	0.587, 0.381∼0.898	21, 5.2	0.003	0.474, 0.287∼0.778
TT	4, 0.7	7, 1.6	0.124	2.544, 0.736∼8.76	6, 1	0.466	1.598, 0.448∼5.684	4, 1	0.552	1.519, 0.375∼6.118
Per T allele	72, 5.9	57, 6.7	0.442	1.151, 0.807∼1.65	49, 4.2	0.069	0.709, 0.491∼1.027	29, 3.6	0.020	0.596, 0.387∼0.931
rs724226			0.034			8×10^−5^			0.678	
GG	467, 75.6	307, 71.2	0.116	1.25, 0.95∼1.645	395, 68.1	0.004	1.45, 1.123∼1.867	309, 75.9	0.897	0.979, 0.731∼1.319
GA	135, 21.8	100, 23.2	0.604	1.078, 0.801∼1.45	140, 24.1	0.346	1.14, 0.865∼1.495	84, 20.6	0.645	0.931, 0.689∼1.265
AA	16, 2.6	24, 5.6	0.013	2.223, 1.166∼4.23	45, 7.8	5×10^−5^	3.167, 1.772∼5.662	14, 3.4	0.429	1.337, 0.646∼2.774
Per A allele	167, 13.5	148, 17.2	0.021	1.329, 1.047∼1.684	230, 19.8	3×10^−5^	**1.579, 1.271∼1.964**	112, 13.8	0.873	1.024, 0.786∼1.323
rs2189814			0.254			0.045			0.643	
CC	292, 47.4	185, 43.1	0.172	1.193, 0.924∼1.527	246, 42.2	0.070	1.233, 0.982∼1.55	202, 49.6	0.485	0.913, 0.708∼1.177
CT	255, 41.4	184, 42.9	0.63	1.064, 0.827∼1.369	246, 42.2	0.779	1.036, 0.819∼1.3	166, 40.8	0.846	0.976, 0.756∼1.26
TT	69, 11.2	60, 14	0.178	1.293, 0.893∼1.866	91, 15.6	0.025	1.47, 1.05∼2.052	39, 9.6	0.409	0.841, 0.552∼1.276
Per T allele	393, 31.9	304, 35.4	0.092	1.171, 0.977∼1.404	428, 36.7	0.013	1.239, 1.05∼1.464	244, 30	0.358	0.911, 0.75∼1.106
rs917071			0.980			0.600			0.914	
CC	374, 60.4	259, 59.8	0.844	1.026, 0.797∼1.317	364, 62.3	0.497	0.926, 0.728∼1.161	249, 60.9	0.883	0.98, 0.762∼1.269
CT	211, 34.1	150, 34.6	0.852	1.022, 0.793∼1.33	184, 31.5	0.341	0.892, 0.701∼1.136	140, 34.2	0.962	1.009, 0.776∼1.307
TT	34, 5.5	24, 5.5	0.972	1.009, 0.591∼1.728	36, 6.2	0.619	1.133, 0.701∼1.835	20, 4.9	0.672	0.887, 0.501∼1.557
Per T allele	279, 22.5	198, 22.9	0.860	1.016, 0.833∼1.252	256, 21.9	0.715	0.968, 0.796∼1.167	180, 22	0.777	0.97, 0.784∼1.196
rs2237558			0.996			0.654			0.661	
GG	395, 63.9	275, 63.7	0.932	1.007, 0.782∼1.303	377, 65	0.695	0.957, 0.748∼1.212	268, 65.8	0.527	0.916, 0.711∼1.198
GA	199, 32.2	140, 32.4	0.944	1.01, 0.774∼1.315	186, 32.1	0.961	0.996, 0.78∼1.262	127, 31.2	0.737	0.959, 0.728∼1.246
AA	24, 3.9	17, 3.9	0.966	1.017, 0.536∼1.91	17, 2.9	0.365	0.747, 0.401∼1.407	12, 2.9	0.426	0.752, 0.377∼1.518
Per A allele	247, 20	174, 20.1	0.930	1.008, 0.817∼1.259	220, 19	0.529	0.934, 0.768∼1.144	151, 18.6	0.422	0.913, 0.731∼1.143
rs6947784			0.595			0.651			0.261	
GC	431, 70.1	289, 67.5	0.379	1.122, 0.865∼1.472	411, 70.5	0.875	0.978, 0.768∼1.256	289, 71	0.751	0.952, 0.731∼1.256
GC	165, 26.8	127, 29.7	0.314	1.148, 0.872∼1.514	159, 27.3	0.863	1.022, 0.795∼1.321	112, 27.5	0.808	1.033, 0.784∼1.376
CC	19, 3.1	12, 2.8	0.789	0.902, 0.438∼1.887	13, 2.2	0.356	0.711, 0.35∼1.461	6, 1.5	0.102	0.467, 0.181∼1.186
Per C allele	203, 16.5	151, 17.6	0.497	1.082, 0.855∼1.362	185, 15.9	0.672	0.951, 0.764∼1.183	124, 15.2	0.443	0.913, 0.71∼1.159
rs2237562			0.540			0.265			0.630	
TT	393, 65.5	263, 63.5	0.518	1.095, 0.843∼1.421	393, 69.7	0.128	0.829, 0.645∼1.06	270, 68.2	0.380	0.89, 0.678∼1.158
TC	181, 30.2	127, 30.7	0.862	1.028, 0.78∼1.345	146, 25.9	0.104	0.81, 0.629∼1.044	112, 28.3	0.523	0.91, 0.693∼1.21
CC	26, 4.3	24, 5.8	0.290	1.357, 0.767∼2.4	25, 4.4	0.934	1.029, 0.585∼1.799	14, 3.5	0.530	0.812, 0.418∼1.574
Per C allele	233, 19.4	175, 21.1	0.343	1.109, 0.897∼1.381	196, 17.4	0.204	0.873, 0.712∼1.076	140, 17.7	0.330	0.894, 0.705∼1.125
rs1468412			0.599			0.273			0.522	
AA	405, 65.4	278, 64.2	0.682	1.051, 0.816∼1.365	406, 69.5	0.130	0.831, 0.651∼1.059	281, 68.7	0.275	0.858, 0.655∼1.121
AT	188, 30.4	131, 30.3	0.967	0.992, 0.76∼1.301	153, 26.2	0.108	0.817, 0.629∼1.044	114, 27.9	0.389	0.891, 0.668∼1.164
TT	26, 4.2	24, 5.5	0.314	1.341, 0.754∼2.362	25, 4.3	0.945	1.02, 0.587∼1.788	14, 3.4	0.528	0.813, 0.414∼1.564
Per T allele	240, 19.4	179, 20.7	0.468	1.087, 0.873∼1.348	203, 17.4	0.205	0.878, 0.71∼1.072	142, 17.4	0.247	0.875, 0.696∼1.099
rs2299225										
TT	564, 92.2	393, 92.7	0.751	0.922, 0.578∼1.486	546, 93.5	0.371	0.822, 0.525∼1.267	376, 92.8	0.687	0.908, 0.565∼1.459
TG+GG c	48, 7.8	31, 7.3			38, 6.5			29, 7.2		
Per G allele	48, 3.9	31, 3.7	0.756	0.931, 0.585∼1.469	40, 3.4	0.371	0.866, 0.565∼1.333	29, 3.6	0.687	0.908, 0.574∼1.453
rs6465088			–			0.061			–	
T	471, 76.1	318, 73.4	0.329	1.156, 0.871∼1.527	461, 78.9	0.237	0.848, 0.644∼1.115	325, 79.5	0.206	0.827, 0.61∼1.114
TG	143, 23.1	115, 26.6	0.200	1.205, 0.904∼1.6	123, 21.1	0.394	0.885, 0.673∼1.166	84, 20.5	0.332	0.863, 0.635∼1.162
GG	5, 0.8	0, 0	–	–	0, 0	–	–	0, 0	–	–
Per G allele	153, 12.4	115, 13.3	0.533	1.09, 0.84∼1.409	123, 10.5	0.160	0.838, 0.653∼1.076	84, 10.3	0.146	0.814, 0.616∼1.075
rs1989796			0.024			0.101			0.497	
TT	354, 57.2	212, 49	0.008	1.391, 1.091∼1.778	309, 52.9	0.136	1.186, 0.943∼1.492	240, 58.7	0.636	0.937, 0.725∼1.213
TC	238, 38.4	194, 44.8	0.039	1.297, 1.017∼1.664	235, 40.2	0.525	1.078, 0.86∼1.359	146, 35.7	0.372	0.891, 0.69∼1.155
CC	27, 4.4	27, 6.2	0.175	1.455, 0.846∼2.525	40, 6.8	0.060	1.607, 0.976∼2.659	23, 5.6	0.357	1.304, 0.736∼2.307
Per C allele	292, 23.6	248, 28.6	0.009	1.304, 1.065∼1.583	315, 27	0.056	1.199, 0.996∼1.436	192, 23.5	0.952	0.996, 0.802∼1.22
rs2282966			0.028			0.387			0.182	
GG	466, 75.4	308, 71.3	0.137	1.235, 0.934∼1.632	450, 77.6	0.374	0.883, 0.679∼1.157	319, 78.4	0.271	0.848, 0.628∼1.142
GA	143, 23.1	107, 24.8	0.542	1.09, 0.824∼1.459	118, 20.3	0.242	0.851, 0.646∼1.122	78, 19.2	0.130	0.788, 0.576∼1.075
AA	9, 1.5	17, 3.9	0.011	2.771, 1.222∼6.278	12, 2.1	0.419	1.432, 0.594∼3.417	10, 2.5	0.245	1.704, 0.686∼4.231
Per A allele	161, 13	141, 16.3	0.034	1.3, 1.02∼1.66	142, 12.2	0.564	0.935, 0.73∼1.19	98, 12	0.511	0.909, 0.7∼1.2
rs7789655										
TT	560, 90.9	379, 87.9	0.119	1.374, 0.923∼2.045	537, 92	0.519	0.876, 0.582∼1.316	377, 92.4	0.402	0.827, 0.52∼1.304
TG+GG c	56, 9.1	52, 12.1			47, 8.0			31, 7.6		
Per G allele	56, 4.5	52, 6	0.119	1.352, 0.914∼1.985	50, 4.3	0.519	0.943, 0.639∼1.391	31, 3.8	0.402	0.831, 0.531∼1.302
rs1716070			0.211			0.838			0.786	
TT	517, 83.5	346, 79.9	0.133	1.274, 0.927∼1.746	494, 84.6	0.613	0.921, 0.674∼1.261	347, 84.8	0.572	0.909, 0.639∼1.276
TC	94, 15.2	83, 19.2	0.089	1.323, 0.959∼1.837	84, 14.4	0.695	0.942, 0.68∼1.293	56, 13.7	0.507	0.881, 0.624∼1.268
CC	8, 1.3	4, 0.9	0.580	0.712, 0.212∼2.377	6, 1	0.668	0.797, 0.272∼2.304	6, 1.5	0.813	1.136, 0.387∼3.306
Per C allele	110, 8.9	91, 10.5	0.213	1.2, 0.898∼1.609	96, 8.2	0.559	0.918, 0.695∼1.225	68, 8.3	0.651	0.934, 0.68∼1.277

a The five *P*-values for each SNP from top to bottom in one column were calculated by 2×3 and 2×2 chi-squared tests orderly based on codominant, dominant for the rare allele, heterosis and recessive for the rare allele, and allelic models of inheritance. – means that the result could not be obtained by chi-squared test as the given genotype frequency is rare.

b OR and 95% CI were calculated by logistic regression analysis with adjustment for demographic factors, including age, gender, education level, unemployment, and life history with family. Statistically significant results with an FPRP value <0.2 are marked in bold.

c The frequency of the homozygote of the minor allele is very rare, and this allele was merged with the heterozygote for a 2×2 chi-squared test.

Sz.: schizophrenia, H.D.: heroin dependence, Dep.: depression.

A comparison between the patients with schizophrenia or depression and the controls revealed significantly different genotypes and/or allele distributions of several SNPs (Table 1). However, these results turned out to be spurious because the FPRPs for these associations between the SNPs and schizophrenia was larger than 0.2 when a prior probability of 0.01 was assigned to detect an OR of 1.5 or 0.67.

**Figure 3:**
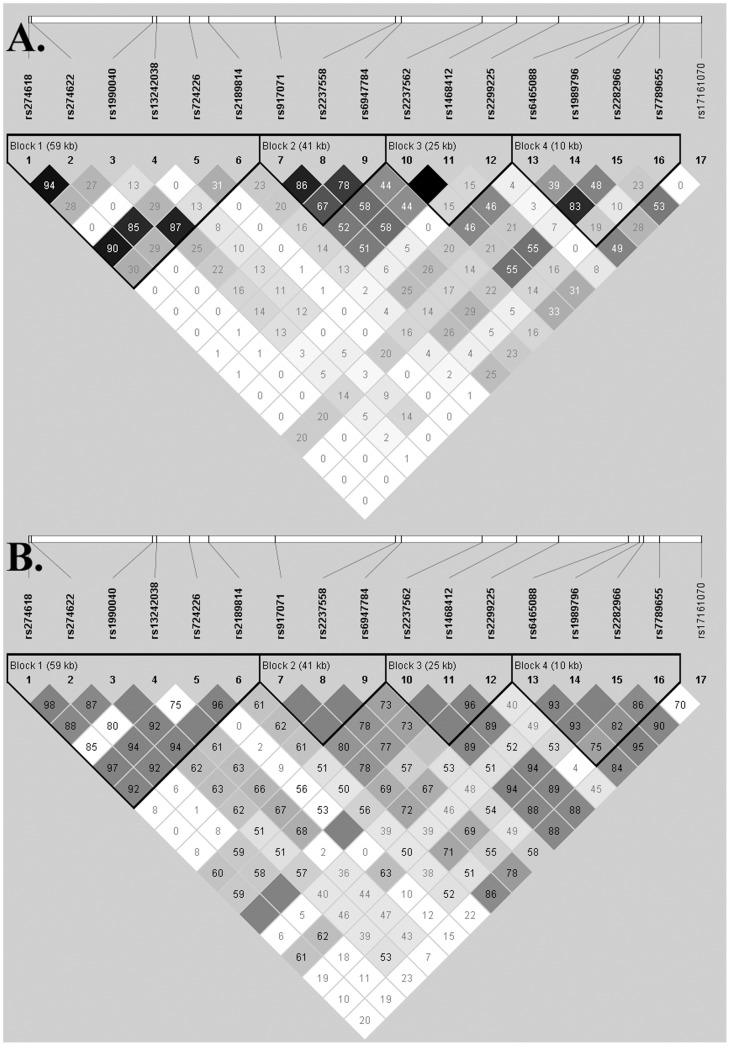
Linkage disequilibrium (LD) plot of 17 *GRM3* SNPs in healthy controls. In figure A, the r^2^ × 100 value corresponding to each SNP pair is shown in the square; black squares indicate r^2^ = 1, i.e., complete LD; white squares indicate r^2^ = 0; shaded grey squares indicate 0< r^2^<1. In figure B, D′ × 100 value corresponding to each SNP pair is shown in the square; white squares indicate D′ <1 and LOD <2; blue squares indicate D′ = 1 and LOD <2; shaded red squares indicate D′ <1 and LOD ≥2; bright red squares indicate D′ = 1 and LOD ≥2.

Because the two positive SNPs associated with HD are in the first haplotype block, which covers the 5′ flanking region of *GRM3* (Fig. 3), the haplotypes of six SNPs (rs274618, rs274622, rs1990040, rs13242038, rs724226, and rs2189814) in this block were further evaluated to determine whether they affect the risk of HD. The frequencies of these haplotypes and the results of an association analysis are shown in Table 2. A haplotype including the minor alleles of rs274618 and rs274622, (T-C-A-C-A-T) was over-represented in heroin addicts (*P* = 0.00001) compared with controls. This haplotype was associated with an increased risk for HD (OR = 1.668, 95% CI: 1.335-2.084) relative to other haplotypes. The FPRP for the association between the T-C-A-C-A-T haplotype and HD was 0.004 given a prior probability of 0.01 to determine an OR of 1.5 or 0.67.

**Table 2:** The frequencies of haplotypes in block 1 and their association with HD.

	SNPs						HD		Controls		Statistics	
							(n = 1168)		(n = 1232^a^ )			
Haplotype^b^	1	2	3	4	5	6	N	%	N	%	*P*-value^c^	OR, 95% CI^d^
1	A	T	G	C	G	G	717	61.4	821	66.6	0.007	0.794, 0.670∼0.940
2	T	C	A	C	A	T	222	19.0	152	12.3	0.00001	**1.668, 1.335∼2.084**
3	A	T	A	C	G	T	135	11.6	149	12.1	0.127	1.208, 0.946∼1.553
4	A	T	A	T	G	T	50	4.3	67	5.4	0.188	0.779, 0.536∼1.133

a Genotyping of several loci in several people failed, and their haplotypes were not determined.

b SNPs 1–6 that comprise a haplotype are: in order, rs274618, rs274622, rs1990040, rs13242038, rs724226, rs2189814. Haplotypes with a frequency <0.01 were excluded.

c *P*-value was calculated by 2×2 chi-squared tests (carrier with a given haplotype vs. non-carrier ).

d OR and 95% CI were calculated by logistic regression analysis with adjustment for demographic factors, including age, gender, education level, unemployment, and life history with family. Statistically significant results with an FPRP value <0.2 are marked in bold.

## Discussion

In the present study, we carried out a genetic association analysis between polymorphisms in *GRM3* and three types of mental disease: schizophrenia, major depression, and HD. We found that *GRM3* polymorphisms modulate the susceptibility to HD but do not significantly influence the risk of schizophrenia or depression. The minor alleles of two SNPs near the 5′ end of *GRM3* (rs274618 and rs274622) were markedly associated with almost 60% greater risk of HD compared with the major alleles. In addition, a haplotype consisting of the minor alleles of the two aforementioned SNPs was significantly associated with a 66.7% greater risk of HD. Moreover, the minor alleles of the two aforementioned SNPs also predicted a markedly shortened DTFUD. This study provides the first evidence that polymorphisms in *GRM3* affect the susceptibility to HD.

The notable association between *GRM3* and HD suggests that mGluR3 may play an important role in the pathogenesis of HD, and this result is consistent with several findings from animal studies. Previous studies using pharmacological intervention with agonist revealed that activation of mGluR 2/3 attenuates self-administration and subsequent relapse to various drugs of abuse [37], [38], [39], [40], [41], [42] and suggest that these receptors negatively regulate the reward and reinforcing effects provided by the drug. Specific agonists and antagonists for each subtype of group II mGluRs are unavailable; therefore, the role of each individual receptor in addiction development should be studied further by other approaches. Morishima et al. reported that mGluR2 knockout mice have increased locomotor sensitization and conditioned place reference induced by cocaine, suggesting an increased reward value of cocaine in the absence of mGluR2 signaling [50]. Our results suggest that mGluR3 may also play a potential role in addiction development.

LD analysis revealed that strong LD existed among the two SNPs that displayed robust association with HD. Moreover, the risk alleles of these SNPs were always present in the same chromosomal location after recombination and were transmitted to offspring as a haplotype. This haplotype within the first block conferred a HD risk similar to each risk allele. These findings suggest that the functional variants for HD may be within the first haplotype block and in strong LD with the two risk SNPs. Another possibility is that one of these risk SNPs may be the functional SNP. Among the risk SNPs, rs274622 is more likely to be the functional SNP for HD. This SNP has been reported to be associated with negative symptom improvement during olanzapine treatment in patients with schizophrenia [9] and phonetic magnetic mismatch field (MMF) recorded by magnetoencephalography in the bilateral auditory cortices of healthy volunteers [51]. More importantly, the T allele of rs274622, which resides in the “T” of a CCAAT box in the *GRM3* promoter region [52], may influence gene expression. Accordingly, people with the rs274622 T/T genotype exhibit significantly decreased MMF strengths in auditory cortices of both hemispheres compared with C/C or C/T carriers [51]. Schizophrenic patients with the rs274622 T/T genotype have significantly smaller improvement in negative symptoms when treated with olanzapine than those with the C/C or C/T genotype [9]. Therefore, this SNP may be functionally relevant to glutamate-mediated synaptic plasticity.

A potential mechanism for addiction development that is supported by increasing evidence is the drug-induced enduring imbalance between synaptic and nonsynaptic glutamate in NAc, termed glutamate homeostasis [7]. Briefly, the imbalance in glutamate homeostasis causes changes in neuroplasticity that impair communication between the prefrontal cortex (PFC) and NAc and contribute to an inability of the PFC to control drug-seeking. Accordingly, an individual’s potential to resist a drug-induced imbalance in glutamate homeostasis reflects variability in the vulnerability to addiction. Accumulating evidence indicates that mGluR2 and mGluR3 are important modulators for synaptic glutamate release by activation of presynaptic K+ channels, inhibition of presynaptic Ca2+ channels, or direct interference with vesicular release [14]. The neuroadaptations in mGluR 2/3 have also been observed in various drugs of abuse [29], [30], [31], [32], [33], [34], [35], suggesting that mGluR 2/3 may mediate the way in which drugs of abuse induce an imbalance in glutamate homeostasis. Additionally, activation of mGluR 2/3 prevents the overflow of glutamate and dopamine in NAc of rats sensitized to amphetamine [53], attenuates the reward effect of nicotine and cocaine [37], [38], and attenuates the antagonism of mGluR 2/3-facilitated, drug-induced glutamate release in NAc after chronic cocaine self-administration [54]. The regulatory effects of mGluR3 on synaptic glutamate release in PFC and NAc may be the biological foundation for the influence of *GRM3* polymorphisms on HD vulnerability. The rs274622 SNP, or other functional SNPs within *GRM3* haplotype block 1 and in strong LD with the aforementioned risk SNPs for HD, may decrease the ability of mGLuR3 to regulate the glutamate balance in the PFC-NAc circuit during chronic heroin use by affecting receptor expression regulation or function and up-regulate the susceptibility to uncontrolled and compulsive heroin use. A notable limitation of our study is lack of replication study. Also, we failed to obtain the findings on the relationship between *GRM3* polymorphisms and heroin dependence from other genetic association studies. Our results are provisional awaiting replication. Future research from large-sample genetic analyses and neuro-imaging studies need to be performed to replicate our findings, clarify the possible roles of these risk SNPs in the control of mGluR3 expression, and identify their potential influence on the regulation of synaptic glutamate balance by mGluR3 under heroin stimulation.

As a replication study, our data fail to find significant associations of *GRM3* with schizophrenia and depression. The relatively large coverage of *GRM3* in our study (17 SNPs) extends previous research that only investigated several SNPs (The investigated SNPs, population, and sample size in previous study are summarized in the Table S1). Meanwhile, to avoid spurious results from multiple studies, we applied a more comprehensive approach (FPRP) to evaluate statistical significance. Unlike traditional methods based on the *P* value alone, FPRP is calculated considering three elements: the observed *P* value, the prior probability that the association between the genetic variant and the disease is real, and the statistical power of the test [48]. In our study, the minor alleles of rs274618 and rs274622 were the most promising risk variants for schizophrenia with the minimum *P* values (*P = *0.010∼0.013). Nevertheless, these alleles were excluded because their FPRPs were greater than 0.4 given a prior probability of 0.01 and an OR of 2. Moreover, these alleles would also be excluded by Bonferroni correction, as 0.007 is greater than the adjusted statistical significance of 0.05/17. In two previous studies, rs1468412 and rs2299225 showed significant association with schizophrenia in Japanese and eastern Chinese populations, respectively [18], [19]. Multiple factors likely contributed to the variable results and include the homogeneity in the cohorts, sample sizes, ethnic background-specific effects, epistasis and gene-environment effects. FPRPs for the associations between schizophrenia and rs1468412 and rs2299225 in the previous studies are greater than 0.5 (prior probability = 0.01) and 0.2 (prior probability = 0.1) to detect an OR of 1.5 according to the computational method described by Wacholder et al. [48]. In sum, our results agree with several previous studies [20], [21], [22], [23], [24] and do not support *GRM3* as a susceptibility gene for schizophrenia. In a recent study, rs6465084 was found to be associated with depression in a Japanese population [10]. However, this SNP was not effectively genotyped in our pilot multi-loci analysis and was not included in further investigation. Our 17 selected SNPs exhibited similar distributions between the patients with depression and the controls. Thus, the current data do not provide significant support for the hypothesis that *GRM3* is a susceptibility gene for depression. If *GRM3* is a true susceptibility gene for depression, its effect on the disease risk is weak or the susceptibility alleles are only in weak LD with the markers we tested.

## Supporting Information

Tables S1 **The summary of previous association studies of** ***GRM3*** **polymorphisms with schizophrenia and mood disorders.**
(DOC)
