# Supplementary material for: Metabotropic Glutamate Receptor 3 Is Associated with Heroin Dependence but Not Depression or Schizophrenia in a Chinese Population
Source: PLoS One. 2014 Jan 31;9(1):e87247. doi: 10.1371/journal.pone.0087247 (PMC3909071; doi:10.1371/journal.pone.0087247)
Supplement: Tables S1 — The summary of previous association studies of GRM3 polymorphisms with schizophrenia and mood disorders. (DOC) [file pone.0087247.s001.doc]

Supplementary Table S1 The summary of previous association studies of *GRM3* polymorphisms with schizophrenia and mood disorders

| Mental Disorder | Population | Sample size (cases/controls or families) | SNP selection | Significantly associated SNPs | Reference |
| --- | --- | --- | --- | --- | --- |
| MDD | Japanese | 325/802 | rs6465084 | rs6465084 | Tsunoka, et al. 2009 [10] |
| BP | Japanese | 155/802 | rs6465084 | None | Tsunoka, et al. 2009 [10] |
| BP | South African | 191/188 | rs6465084 | rs6465084 | Dalvie, et al. 2010 [11] |
| Sz | Japanese | 1916/1915 | rs1468412, rs2299225, rs274622 | None | Albalushi, et al. 2008 [24] |
| Sz | UK and Ireland | 674/716 | rs187993, rs13242038, rs917071, rs6465084, rs2228595, rs1468412, rs7804100 | None | Norton, et al. 2005 [22] |
| Sz | Chinese | 752/752 | rs274622, rs2282958, rs724226,  rs757656, rs2237562, rs1468412,  rs2299225 | rs2299225 | Chen, et al. 2005 [19] |
| Sz | European American | 217/136;  128 families | rs6465084 | rs6465084 | Egan, et al. 2004 [8] |
| Sz | Japanese | 100/100 | rs274622, rs724226, rs917071, rs1468412, rs1989796, rs1476455 | rs1468412 | Fujii, et al. 2003 [18] |

MDD: major depressive disorder; BP: bipolar disorder; Sz: schizophrenia
